# Characteristics and predictors of clinical outcome in patients with pleural effusions caused by heart, liver and renal failure: results from the ERS International Multicentre Pleural Research Collaborative (IMPACT) registry

**DOI:** 10.1183/23120541.00169-2025

**Published:** 2025-12-01

**Authors:** Hugh Welch, Steven Walker, Jordy Kerkhoff, Julius Janssen, Silvia Bielsa, Carmen Civit, Jose M. Porcel, Katrine Fjaellegaard, Jesper Petersen, Uffe Bodtger, Elzbieta M. Grabczak, Mohamed Ellayeh, Dinesh Addala, John M. Wrightson, Najib M. Rahman, Karl A. Jackson, Emilia I. Pellas, Irfan I. Khan, Muhammed T. Chohan, Avinash Aujayeb, Gonzalo Labarca, Inderdeep Dhaliwal, Michael A. Mitchell, Sumit Chatterji, Ales Rozman, Mateja Marc-Malovrh, Stavros Anevlavis, Marlos Froudrakis, Federico Mei, Paul White, Nick Maskell, Jane Shaw, Rahul Bhatnagar

**Affiliations:** 1 Academic Respiratory Unit, Bristol Medical School: Translational Health Sciences, University of Bristol, Bristol, UK; 2 Southmead Hospital, North Bristol NHS Trust, Bristol, UK; 3 Department of Pulmonary Diseases B70, Canisius Wilhelmina Ziekenhuis, Nijmegen, the Netherlands; 4 Pleural Medicine Unit, Department of Internal Medicine, Arnau de Vilanova University Hospital, IRB Lleida, Lleida, Spain; 5 Department of Respiratory Medicine, Zealand University Hospital Næstved/Roskilde, University of Southern Denmark, Institute of Regional Health Research, Odense, Denmark; 6 Respiratory Department, University Clinical Center, Warsaw, Poland; 7 Department of Chest Medicine, Mansoura University Faculty of Medicine, Mansoura, Egypt; 8 Oxford Centre for Respiratory Medicine, Oxford University Hospitals NHS Foundation Trust, Oxford, UK; 9 Oxford NIHR Biomedical Research Centre, Oxford, UK; 10 Respiratory Department, Northumbria Healthcare Foundation Trust, Cramlington, UK; 11 Division of Internal Medicine, Complejo Asistencial Dr Víctor Ríos Ruiz, Los Angeles, Chile; 12 Department of Respiratory Diseases, School of Medicine, Pontificia Universidad Católica de Chile, Santiago, Chile; 13 Department of Medicine, Schulich School of Medicine and Dentistry, Western University, London, ON, Canada; 14 Pleural Service, Sheba Academic Medical Center, Tel Aviv, Israel; 15 University Clinic Golnik, Golnik, Slovenia; 16 Democritus University of Thrace, University Hospital of Alexandroupolis, Alexandroupolis, Greece; 17 Respiratory Diseases Unit, Department of Internal Medicine, Azienda Ospedaliero-Universitaria, Ospedali Riuniti, Ancona, Italy; 18 University of the West of England, Bristol, UK; 19 DSI/NRF Centre of Excellence for Biomedical Tuberculosis Research, South African Medical Research Council Centre for Tuberculosis Research, and Division of Immunology and Division of Pulmonology, Faculty of Medicine and Health Sciences, Stellenbosch University, Cape Town, South Africa

## Abstract

**Introduction:**

Pleural effusions caused by organ dysfunction are the commonest pleural disease and account for a huge healthcare burden. Previous work has demonstrated poor survival rates, but there is still uncertainty about determinants of prognosis. This study describes the characteristics and risk factors for poor outcomes in patients with pleural effusion secondary to organ failure in an international cohort.

**Methods:**

The European Respiratory Society International Multicentre Pleural Research Collaborative (IMPACT) registry includes an international retrospective study of patients with effusions secondary to heart, liver or renal failure, collected from 10 countries in Europe and North and South America between 2019 and 2021. The data were analysed for associations between baseline patient characteristics and key clinical outcomes. Descriptive data were collected on treatments and complications.

**Results:**

A total of 755 patients contributed data. Overall, 85.2% of effusions were classified as transudates by Light's criteria. 42% of effusions were bilateral. One-year mortality rates were 46% in renal, 35% in hepatic and 33% in cardiac effusions. Increased mortality was observed in neutrophil-predominant effusions (HR 2.001, 95% CI 1.202–3.349, p=0.008), with age (HR 1.013, 95 CI 1.002–1.024, p=0.02) and with N-terminal pro-brain natriuretic peptide >450 pg·mL^−1^ (HR 1.508, 95% CI 1.191–1.911) in patients with cardiac failure. Therapeutic thoracentesis was the most frequently employed pleural intervention; indwelling pleural catheter use was rare and associated with higher pleural infection rates than thoracentesis.

**Conclusion:**

This study identifies prognostic factors in an international cohort of patients with transudative pleural effusions. Identification of these risk factors may support treatment approaches in a global population.

## Shareable abstract

**This large, multicentre, international, observational study of pleural effusions in patients with heart, liver or renal failure identified several key prognostic factors, such as a neutrophil predominance and high NT-proBNP, that can aid decision-making** https://bit.ly/3EZL7v5

## Introduction

Transudative pleural effusions develop when the systemic hydrostatic or oncotic factors influencing the formation or absorption of pleural fluid are altered so that pleural fluid accumulates. It is an important clinical condition, which often persists for years, causes high symptom burden and poses diagnostic challenges [1]. In the USA, the incidence is 252 out of 100 000, accounting for 75% of healthcare spending on pleural effusions [2–4]. Two prospective single-centre studies demonstrated that pleural effusions resulting from organ dysfunction are associated with similar mortality rates to those of some malignancies [5, 6]. Identification of poor prognostic factors would help guide patient management. The International Multicentre Pleural Research Collaborative (IMPACT) is a European Respiratory Society (ERS) clinical research collaboration [7]. A project from this network aimed to identify key prognostic factors and provide descriptive data regarding treatments and complications in patients with pleural effusion secondary to organ failure.

## Methods

### Data collection

Retrospective cohort data were collected from 12 centres in 10 countries between 2019 and 2021. Where necessary, local ethical approval was gained to contribute anonymised data collected as part of routine care or held in local research repositories. Centres were requested to screen their local dataset(s) for adult patients with a confirmed diagnosis of pleural effusion related to cardiac, renal and hepatic failure between 2004 and 2020. In patients with effusions potentially related to a combination of these causes, centres were asked to categorise patients according to the dominant pathology. Anonymised data were entered by participating sites directly into a REDCap database, hosted at the University of Bristol [8]. Data points of interest included underlying diagnoses, demographics, clinical and radiological features, procedural and interventional records, and records of pleural intervention complications. Pleural fluid cytological predominance was defined as per British Thoracic Society guidelines (>50%) [9].

### Statistical analysis

Analysis was performed using SPSS Statistics 28.0 (SPSS, Chicago, IL, USA). Descriptive and inferential statistics are presented. The mean±sd is reported for parametric data and the median (interquartile range (IQR)) for non-parametric data. The Mann–Whitney U test was used to compare medians and one-way ANOVA for continuous variables. Mortality rates and Kaplan–Meier curves calculated for survival probability are presented for variables identified using both Kaplan–Meier and Cox regression methods. To avoid excessive data loss, available case analysis was used. Unless otherwise stated, percentages are based on the total number of patients in each subtype. Where possible, discrepancies in measurement units were corrected into standard form. A subgroup analysis of the cardiac dataset was performed to identify variables that exert significant effects on patient mortality using logistic regression and purposeful selection [10]. A p-value of <0.05 was considered significant.

## Results

**FIGURE 1:**
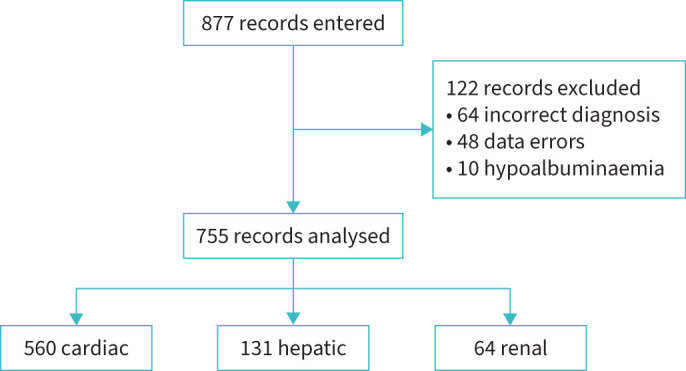
Flowchart of records in the database.

In total, 877 individual case records were submitted with 755 cases suitable for analysis (figure 1). Of these, 122 cases were removed due to diagnostic uncertainty, mislabelling or insufficient data entry, including 10 cases in which the effusion was most likely due to hypoalbuminaemia. The number of records contributed by site is shown in supplementary table S1. The median length of follow-up was 481 days (IQR 130–1178 days).

### Baseline characteristics

Complete demographic and characteristic data at presentation are shown in table 1. The cohort was multimorbid, with a high incidence of multisystem disease. Dyspnoea (89%) and cough (32%) were the most commonly reported symptoms (supplementary table S2). Pleuritic pain and fever were reported by 7.0% and 7.2%, respectively; however, only 0.9% reported both these symptoms. There were no reported cases of infection in either group, and neutrophil predominance was identified in seven out of 53 patients with pleuritic pain and six out of 55 patients with fever. Both symptoms were reported more frequently by patients with transudative effusions (pain: 34 out of 53 patients; fever: 42 out of 55 patients).

**TABLE 1:** Characteristics of patients with effusions related to cardiac, renal and hepatic disease

	Total	Cardiac	Renal	Hepatic	p-value
**Participants (n)**	755	560	64	131	
**Male**	460 (60.9)	332 (59.3)	44 (68.8)	84 (64.1)	0.246
**Median age (years) (IQR)**	70.0 (54.0–86.0)	79.0 (71.2–86)	75.5 (64.3–81.8)	63.0 (54.0–73.0)	<0.001
**Pre-existing co-morbidities at presentation**					
COPD	120 (15.9)	96 (17.1)	8 (12.5)	16 (12.2)	0.314
Pulmonary embolus	17 (2.3)	16 (2.9)	0	1 (0.8)	0.235
Ischaemic heart disease	218 (28.9)	181 (32.3)	22 (34.4)	15 (11.5)	<0.001
Heart failure	299 (39.6)	256 (45.7)	24 (37.5)	9 (6.9)	<0.001
Hypertension	394 (52.2)	315 (56.3)	30 (46.9)	49 (37.4)	<0.001
Atrial fibrillation	260 (34.4)	231 (41.3)	17 (26.6)	12 (9.2)	<0.001
Cerebrovascular disease	82 (10.9)	70 (12.5)	4 (6.3)	8 (6.1)	0.052
Alcoholic liver disease	85 (11.3)	12 (2.1)	4 (6.3)	69 (52.7)	<0.001
Autoimmune liver disease	15 (2.0)	3 (0.5)	1 (1.6)	11 (8.4)	<0.001
Non-alcoholic steatohepatitis	12 (1.6)	3 (0.5)	2 (3.1)	8 (6.1)	<0.001
CKD 1–2	277 (36.7)	191 (34.1)	2 (3.1)	84 (64.1)	<0.001
CKD 3–4	282 (37.4)	226 (40.4)	26 (40.6)	30 (22.9)	0.225
CKD 5	24 (3.2)	0	21 (32.8)	3 (2.3)	0.159
Type 1 diabetes	11 (1.5)	5 (0.9)	2 (3.1)	4 (3.1)	0.053
Type 2 diabetes	225 (29.8)	168 (30)	18 (28.1)	39 (29.8)	0.972
Rheumatoid/connective tissue disease	26 (3.4)	19 (3.4)	3 (4.7)	4 (3.1)	0.550
Hypothyroidism	32 (4.2)	22 (3.9)	0	10 (7.6)	0.131
**Exposures (n/N (%))**					
Smoking history^#^	295/514 (57.4)	206/377 (54.6)	23/33 (69.7)	66/104 (63.5)	0.002
Asbestos exposure^#^	31/200 (15.5)	22/126 (17.4)	3/26 (11.5)	6/48 (12.5)	0.001

Data are presented as n (%), unless otherwise indicated. CKD: chronic kidney disease stage (according to the Kidney Disease: Improving Global Outcomes guidelines for classification of glomerular filtration rate); IQR: interquartile range. ^#^: the denominator represents the number of records with data on this exposure.

### Effusions due to cardiac failure

The median age of patients with pleural effusions secondary to heart failure was 79.0 years (71.2–86.0 years) (table 1). The commonest ECG finding was atrial fibrillation (n=211, 37.7%) (supplementary table S3). Left ventricular hypertrophy (n=16, 2.9%), left bundle branch block (n=26, 4.6%) and right bundle branch block (n=19, 3.4%) were less common ECG findings. In patients with numerically reported left ventricular ejection fraction (LVEF) values (n=254), 79 patients (31.1%) had heart failure with reduced ejection fraction (HFrEF), whereas 136 patients (53.5%) had heart failure with preserved ejection fraction (HFpEF) [11]. Mitral valve dysfunction was the commonest valvular pathology, reported in 216 patients (63.0%). Moderate to severe chronic kidney disease (stage 3–4) was a common comorbidity, in 40% of patients.

Of the cardiac effusion group, chest X-ray demonstrated 192 (34.4%) unilateral right-sided effusions and 98 (17.5%) unilateral left-sided effusions. A total of 268 (48.0%) had bilateral effusions, of which 76 (28.4%) were left-predominant and 126 (47.0%) were right-predominant (supplementary table S4). Most effusions (n=699, 79.1%) occupied <50% of the hemithorax at presentation. The cardiac pleural effusions were characterised as transudative by Light's criteria in 326 patients (86.5%) with matched serum and pleural fluid data. Cardiac failure in the exudative group was confirmed by the presence of raised N-terminal pro-brain natriuretic peptide (NT-proBNP) values and/or evidence of cardiac dysfunction on echocardiogram and the exclusion of alternate pathologies such as infection or malignancy. The cytological makeup of cardiac effusions was split between mesothelial/macrophage predominant effusions (n=181, 50.4%) and lymphocytic collections (n=147, 40.9%) (table 2). A small number (n=31, 8.6%) were neutrophilic. The median neutrophil:lymphocyte ratio was 0.22 (IQR 0.06–0.78). A comparison of features of transudative and exudative cardiogenic pleural effusions is detailed in supplementary table S10.

**TABLE 2:** Biochemical and cytological features by pleural effusion subtype

	Total	Cardiac	Renal	Hepatic	p-value
**Biochemical values**					
Total with data (n)	466	323	38	105	
pH	7.48 (7.42–7.52)	7.48 (7.42–7.52)	7.42 (7.35–7.47)	7.49 (7.43–7.53)	0.003
Glucose (mmol·L^−1^)	129.6 (111.6–156.6)	130 (113.0–159.0)	122.5 (102.2–147.6)	126.2 (109.8–154.0)	0.176
Pleural fluid LDH:serum LDH ratio^#^	0.359 (0.261–0.475)	0.365 (0.272–0.474)	0.332 (0.190–0.495)	0.336 (0.231–0.471)	0.375
Protein (g·L^−1^)	20.0 (14.0–28.0)	21.0 (16.0–29.0)	18.5 (12.0–30.0)	18.0 (12.0–23.0)	0.049
Transudates (by Light's criteria)	397 (85.2)	279 (86.4)	28 (73.7)	90 (85.7)	0.182
**Cell predominance**					
Total with data (n)	460	359	30	71	
Lymphocyte	197 (42.8)	147 (40.9)	6 (20.0)	44 (62.0)	<0.001
Mesothelial/macrophage	216 (47.0)	181 (50.4)	19 (63.3)	16 (22.5)	<0.001
Neutrophil	46 (10.0)	31 (8.6)	5 (16.7)	10 (14.1)	0.069
Eosinophil	1 (0.2)	0	0	1 (1.4)	0.532
Pleural fluid neutrophil:lymphocyte ratio	0.22 (0.07–0.71)	0.22 (0.06–0.78)	0.45 (0.11–1.10)	0.15 (0.06–0.53)	0.944

Data are presented as n (%) or median (IQR), unless otherwise indicated. LDH: lactate dehydrogenase. ^#^: LDH values reported with varying units, therefore LDH reported as ratio of serum and pleural fluid values.

### Effusions due to hepatic failure

The median age of patients with pleural effusions secondary to hepatic failure was 63.0 years (54.0–73.0 years) (table 1). Cirrhosis was identified in 90 of 131 patients in the hepatic group (68.7%), with ultrasonographic evidence of portal hypertension present in 65 (49.6%) (supplementary table S5). Ascites was reported in 80 (61.1%). In the non-cirrhotic group, (non-cirrhotic) portal hypertension, acute liver injury, steatohepatitis and chronic liver disease secondary to pancreatitis were reported. Only 22 patients (16.8%) had bilateral pleural effusions, 91 (69.5%) had right-sided effusions and 18 (13.7%) had left-sided effusions (supplementary table S4). Most patients (55%) had effusions which occupied <50% of hemithorax. The hepatic pleural effusions were characterised as transudative as per Light's criteria in 90 patients (87.4%) with matched serum and pleural fluid data. The cytological makeup was predominantly lymphocytic (62.8%). The median neutrophil:lymphocyte ratio was 0.15 (IQR 0.06–0.53).

### Effusions due to renal failure

The median age of patients with pleural effusions secondary to renal failure was 75.5 years (64.3–81.8 years). The majority of patients (n=47, 96%) had ≥stage 3 chronic kidney disease (table 1). In total, 27 patients (42.2%) had bilateral pleural effusions, 23 (35.9%) had right-sided effusions and 14 (21.9%) had left-sided effusions (supplementary table S4). 21 (71.0%) had effusions that occupied <50% of hemithorax. Renal pleural effusions were characterised as transudates as per Light's criteria in 28 patients (77.8%) with matched serum and pleural fluid data. The cytological makeup was predominantly mesothelial/macrophage (63.3%). The median neutrophil:lymphocyte ratio was 0.45 (IQR 0.11–1.10).

### Prognostic factors

**FIGURE 2:**
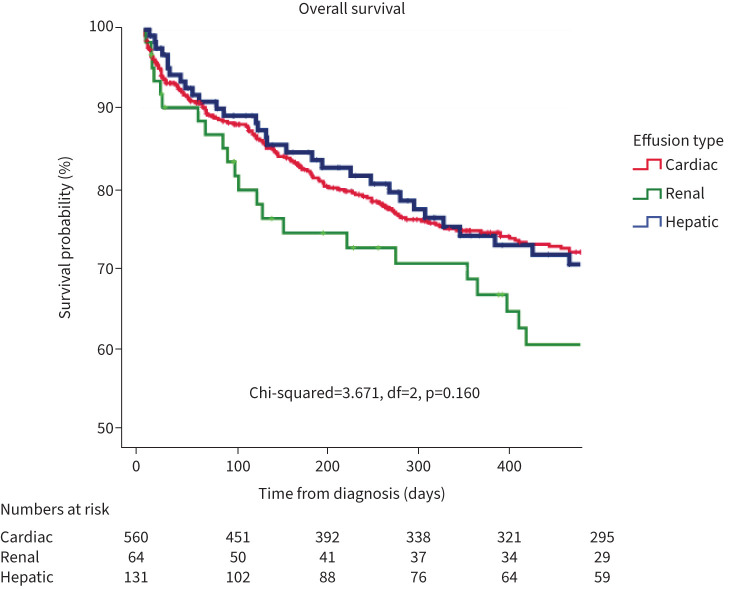
Kaplan–Meier survival curve for cardiac, renal and hepatic effusions. df: degrees of freedom.

**FIGURE 3:**
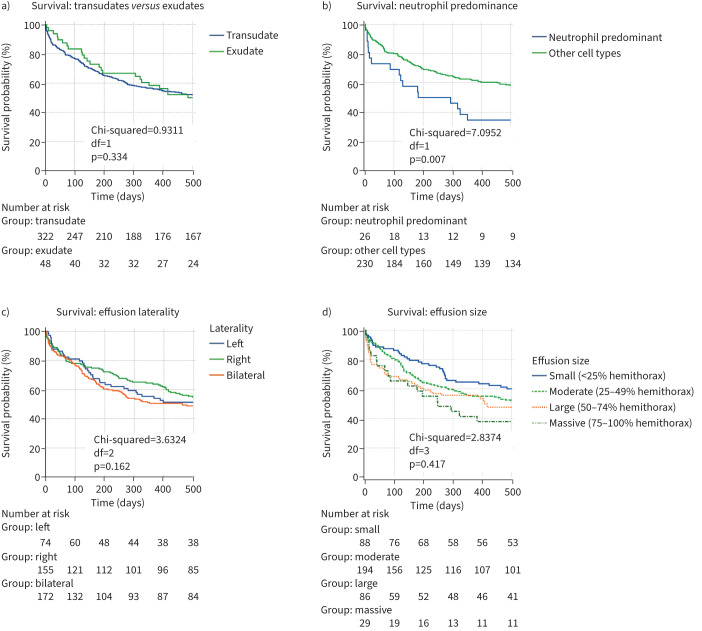
Kaplan–Meier curves for all effusion types for a) transudates *versus* exudates, b) neutrophil predominance, c) effusion laterality and d) effusion size. df: degress of freedom.

Kaplan–Meier curves for overall survival in each group were calculated and are shown in figure 2. There were no statistically significant differences in survival between groups. Curves were also calculated for variables including transudate/exudate, neutrophil predominance, effusion laterality and effusion size (figure 3). Overall, the only factor associated with a worse survival was neutrophil predominance in the pleural fluid (HR 2.001, 95% CI 1.202–3.349, p=0.008). In the subgroup with cardiac effusions, age (HR 1.013, 95% CI 1.002–1.024, p=0.02) and serum NT-proBNP value >450 pg·mL^−1^ (HR 1.508, 95% CI 1.191–1.911, p<0.001) were associated with poorer prognosis. Bilateral effusions were not significantly associated with poorer prognosis (HR 1.114, 95% CI 0.957–1.298, p=0.162). These were associated with higher mean NT-proBNP values (p=0.009). Diastolic dysfunction was associated with improved survival compared to systolic dysfunction (HR 0.738, 95% CI 0.592–0.920, p=0.007). Neither the presence of ischaemic heart disease (HR 1.238, 95% CI 0.963–1.592, p=0.096) nor LVEF ≤50% (HR 1.135, 95% CI 0.967–1.332, p=0.122) were associated with prognosis (supplementary table S6). No statistically significant associations were found between pleural interventions and survival.

### Management of transudative effusions

Loop diuretics were the most frequently used medication in all groups, with high rates of mineralocorticoid antagonist use reported in the hepatic group (supplementary table S7). Beta-blockers were used with comparable frequency in all groups, with lower rates of angiotensin-converting enzyme inhibitors and angiotensin-II-receptor blockers in the hepatic group. In the cardiac effusion group, interventional cardiac procedures were reported rarely (pacemaker insertion 0.9%, implantable defibrillators 1.1%, resynchronisation devices 0.4%) (supplementary table S8). Approximately one third (n=22, 34.3%) of renal effusion patients were undergoing haemo- or peritoneal dialysis at presentation. A further 10.9% were subsequently initiated on dialysis, of which six (9.4%) underwent haemodialysis. Hepatic effusions were managed with paracentesis in 76 cases (58.0%), and trans-hepatic portosystemic shunt insertion was undertaken in six patients (4.6%). The pleural interventions undertaken are displayed in table 3. Therapeutic thoracentesis was the most common pleural intervention in all subtypes. Chest tube drainage and indwelling pleural catheter (IPC) insertion were employed less frequently than recurrent thoracentesis in all groups. IPC use was highest in the hepatic group.

**TABLE 3:** Pleural interventions and associated complications in all groups

	Total	Cardiac	Renal	Hepatic	p-value
**Participants (n)**	755	560	64	131	
**Pleural intervention**					
Therapeutic thoracentesis	519 (68.7)	402 (70.8)	28 (43.8)	89 (67.9)	<0.001
1 thoracentesis	170 (22.5)	116 (20.7)	24 (37.5)	30 (22.9)	<0.001
>1 thoracentesis	98 (13.0)	56 (10.0)	10 (15.6)	32 (24.4)	<0.001
Chest tube drainage	74 (9.8)	49 (8.8)	8 (12.5)	17 (13)	0.220
Talc *via* chest tube	9 (1.2)	7 (1.3)	0	2 (1.5)	0.127
IPC	35 (4.6)	17 (3.0)	1 (1.6)	17 (13)	<0.001
Spontaneous pleurodesis with IPC	15 (2.0)	4 (0.7)	1 (1.6)	10 (7.6)	<0.001
Intravenous albumin	26 (3.4)	7 (1.3)	4 (6.3)	15 (11.5)	<0.001
**Complications**					
Pleural infection	37 (4.9)	25 (3.4)	3 (4.7)	9 (6.9)	0.040
IPC-related	7 (0.9)	3 (0.5)	0	4 (3.1)	0.019
Non-IPC related	30 (4.0)	22(3.0)	3 (4.7)	5 (3.8)	0.902
Electrolyte disturbance	67 (8.9)	33 (5.9)	7 (10.9)	27 (20.6)	<0.001
AKI <48 h after thoracentesis	4 (0.5)	0	1 (1.6)	3 (2.3)	0.006
AKI >48 h after thoracentesis	9 (1.2)	1 (0.2)	0	8 (6.1)	<0.001

Data are presented as n (%), unless otherwise indicated. AKI: acute kidney injury; IPC: indwelling pleural catheter.

### Complications with pleural intervention

Rates of non-IPC-related pleural infection were low across all investigated groups, although the highest rates occurred in the hepatic effusion group (table 3). Recurrent therapeutic thoracentesis was not significantly associated with pleural infection (2.4% in recurrent thoracentesis group *versus* 4.4% in the single or no thoracentesis group, p=0.249). Rates of IPC-related infection were higher: three out of 17 (17.6%) in cardiac and four out of 17 (23.5%) in hepatic effusions. Post-procedural electrolyte disturbance and acute kidney injury (AKI) rates were low. They occurred with the greatest frequency in the hepatic effusion group. In hepatic effusion patients, electrolyte disturbance and AKI rates were higher in those given intravenous (*i.v.*) albumin (n=7, 46.7%) compared to those who were not (n=19, 17.4%) (supplementary table S9). This likely reflects the practice of *i.v.* albumin administration in patients with suspected paracentesis-induced circulatory dysfunction.

## Discussion

This study represents the largest reported case series of pleural effusions related to cardiac, renal and hepatic dysfunction, encompassing records from 12 sites across three continents. We have shown that patients presenting with a first diagnosis of an effusion due to heart, liver or kidney failure have a high 1-year mortality rate, emphasising the importance of a symptom-based approach to the management of this condition.

The most frequently occurring symptoms at presentation were cough and dyspnoea. However, small numbers reported pleuritic pain and/or fever. In this dataset, infection was excluded; however, neutrophil-predominant effusions were noted in a minority of both groups. These factors may be suggestive of an inflammatory component in some nonmalignant pleural effusions.

In the cardiac group, comorbidity and multisystem disease rates were high. The mean patient age correlated with the established severe cardiac disease incidence peak of 75 years [5, 12]. Renal patients were predominantly older adults with multisystem disease. Underlying renal disease was frequently advanced, with a high proportion of patients on dialysis at presentation. The hepatic group was the youngest and least morbid, reflecting the earlier onset of hepatic disease, in particular alcoholic liver disease [13–15].

Patients with renal disease-related effusions had a nonsignificant trend towards higher mortality rate over 1 year from pleural effusion diagnosis than the other two groups. The mortality rates in the renal and hepatic groups were similar to those reported in other studies, but the group with cardiac effusions had a lower mortality rate than expected [5, 6]. Data from other studies suggest a 1-year mortality rate with cardiac effusions of 50–53%, whilst our cohort only had 33% mortality at 1 year [5, 6]. A plausible explanation is that patients in this global database may have undergone pleural sampling earlier in their disease than reported elsewhere [5, 6]. Alternatively, this difference could reflect more recent improvements in heart failure management and the resultant reduction in mortality [16].

We showed that older age and higher NT-proBNP levels were significantly associated with mortality in the cardiac pleural effusion group. NT-proBNP, a natriuretic peptide synthesised in response to cardiomyocyte stretch, is a frequently used blood test that is employed as a surrogate marker of cardiac failure [17]. Our finding correlates with the established increase in heart failure mortality in the older population [18, 19].

Over half of patients had unilateral effusions at presentation. This challenges the long-held view that transudative effusions are typically bilateral, suggesting that organ failure should always be considered in the workup of unilateral pleural effusions [20–24]. Right-sided effusions occurred more frequently then left-sided in all groups. Whilst bilateral effusions can be explained by volume overload and oncotic pressures, in effusions with abdominal origin such as hepatic and peritoneal dialysis-related hydrothorax, rightwards predominance may be related to trans-diaphragmatic passage of abdominal fluid through diaphragmatic muscle defects and embryological remnants such as the pneumatoenteric recess and infracardiac bursa, both commonly right-sided [25, 26]. Bilateral and left-sided effusions were associated with similar mortality; however, right-sided effusions are associated with a slightly better prognosis.

Although the majority of effusions were classified as transudates using Light's criteria, between 15% and 20% were exudates. This is consistent with previous literature on effusions caused by major organ failure [21, 22, 27].

Light's criteria were originally designed to be most sensitive for exudates, and it is therefore expected that a proportion of transudative effusions will be misclassified when using these parameters [1, 28]. However, it is also possible that some of the effusions were true exudates. Chronic diuretic use may transform a transudative effusion to an exudate; in this study, diuretic usage rates were higher in the exudative cardiac effusions group [29]. Within the renal group, exudates were thought to be secondary to uraemic pleuritis [30, 31]. There is also the small possibility that some of these exudates represent undiagnosed pleural infection. Patients with advanced organ failure often have immune dysfunction and deficiency (or are treated with immunosuppressants), which predispose them to pleural infection even in the absence of a pleural procedure [32]. Translocation of microorganisms between sterile and nonsterile sites is well known in advanced liver disease and is likely a feature of many other conditions in which there are high circulating levels of proinflammatory cytokines [33].

Our evidence suggests that the pleural fluid neutrophil:lymphocyte ratio might provide further insight into this phenomenon, because the ratio was highest in the renal effusion group, which also had the numerically highest mortality rate (although not statistically significant). The cardiac effusions group was split between mesothelial/macrophage predominant and lymphocyte predominant effusions, supporting the hypothesis of oncotic pressure-related effusion formation rather than neutrophil-driven inflammation [34, 35]. However, even in this group a small number were neutrophil-predominant. The neutrophil:lymphocyte ratio is a well-described marker of inflammation and disease severity in malignant and tuberculous pleural effusions [36–39]. Here, we have reported it for the first time in nonmalignant, noninfectious effusions and demonstrated that it is a predictor of outcome, suggesting that these conditions are associated with a low and chronic level of inflammation which may have a meaningful clinical impact on patients. It is possible that the presence of a neutrophil-predominant effusion may suggest serious undetected pathologies such as infection or pulmonary embolus, although in this dataset these pathologies were excluded by reporting centres.

In the cardiac effusion group, we observed that echocardiogram-identified HFpEF and HFrEF are both associated with pleural effusion, suggesting that clinicians managing cardiac effusions should consider both aetiologies when treating these patients. Established cirrhosis was identified in half of hepatic effusions, with similar rates of portal hypertension and ascites detected. The 40.5% of patients with no ultrasonographically detectable ascites was in keeping with the recognised phenomenon of the rate of ascites generation meeting the trans-diaphragmatic passage rate, resulting in “ascitic” hepatic hydrothorax in the absence of abdominal fluid [40].

Overall procedural complication rates were low, although IPC-related infection rates (17.6–23.5%) were higher than those in a recently published meta-analysis [41]. Interestingly, there was no association between pleural infection and repeated therapeutic thoracentesis in this cohort, contrary to usual teaching.

No significant association was found between pleural interventions and survival in the cardiac group, suggesting that interventions in this cohort may be considered on the basis of symptom palliation rather than survival benefit.

Our study is the largest multicentre case series on organ failure-related pleural effusions and provides the most comprehensive dataset of this complex and under-researched disease group with huge epidemiological importance and healthcare resource utilisation.

This study has several limitations which should be considered by readers. Prior to data collection, exact criteria to define the aetiology of the effusion types were not set, thus the data analysed rely on the interpretation of local clinicians, which may be subjective and potentially variable across centres. The retrospective nature of the study unavoidably introduces selection and recall bias to the dataset. Further to this, data heterogeneity and missing data limit meaningful statistical analysis and the conclusions that may be drawn. Some of the definitions were not objectively verifiable, such as the occurrence of pleural infection after a procedure. Reporting of certain data was not standardised across sites, *e.g* for the LVEF, which may introduce both bias and error. Lastly, the small numbers in the renal and hepatic groups mean that any results must be interpreted with caution.

### Conclusions

This large-scale study has described the characteristics, prognostic factors, management strategies and associated complications of transudative effusions. Information from this study may be used to help inform clinicians, patients and their families regarding treatment decisions and likely outcomes.

## Supplementary material

**Please note:** supplementary material is not edited by the Editorial Office, and is uploaded as it has been supplied by the author. Supplementary material 00169-2025.SUPPLEMENT
