# Supplementary material for: Characteristics and predictors of clinical outcome in patients with pleural effusions caused by heart, liver and renal failure: results from the ERS International Multicentre Pleural Research Collaborative (IMPACT) registry
Source: ERJ Open Res. 2025 Dec 1;11(6):00169-2025. doi: 10.1183/23120541.00169-2025 (PMC12683546; doi:10.1183/23120541.00169-2025)
Supplement: Supplementary file 1 [file 00169-2025.SUPPLEMENT.pdf]

Characteristics and predictors of clinical outcome in patients with pleural effusions caused by heart, liver and renal failure: results from the ERS International Multicentre Pleural Research Collaborative (IMPACT) registry

Supplementary tables

Institution	Total n=755	Cardiac n=560	Renal n=64	Hepatic n=131
Canisius Wilhelmina Ziekenhuis, Netherlands	279	249	15	15
Arnau de Vilanova University Hospital, Spain	205	134	-	71
University Clinical Centre, Poland	67	43	10	14
Southmead Hospital, UK	59	32	14	13
Naestved Hospital, Denmark	58	44	10	4
Northumbria Healthcare NHS Foundation Trust, UK	40	26	6	8
London Health Sciences Centre, Ontario, Canada	20	15	3	2
Complejo Asistencial Dr Victor Rios Ruiz, Chile	17	11	4	2
Sheba Academic Medical Centre, Israel	6	4	2	-
University Clinic Golnik, Slovenia	2	-	-	2
John Radcliffe Hospital, UK	1	1	-	-
University Hospital of Alexandroupolis, Greece	1	1	-	-

Supplementary table S1: Contributing sites to the International Collaborative Effusion Non-Malignant Pleural Effusion study, with total analysable case contributions and contributions split by underlying pathology

Symptom n (%)	Cardiac n=560	Renal n=64	Hepatic n=131	p value
Dyspnoea	502 (89.9)	56 (87.5)	115 (87.8)	0.688
Cough	192 (34.3)	20 (31.3)	33 (25.2)	0.125
Pleuritic Pain	42 (7.5)	6 (9.4)	5 (3.8)	0.245
Weight Loss	40 (7.1)	5 (7.8)	4 (3.1)	0.181
Fever	41 (7.3)	3 (4.7)	11 (9.4)	0.722
Night sweats	4 (0.7)	0	0	1.0

Supplementary table S2: Symptoms at presentation

ECG Features		n (%)
n=560	Atrial Fibrillation	211 (37.7)
	Left Ventricular Hypertrophy	16 (2.9)
	Left Bundle Branch Block	26 (4.6)
	Right Bundle Branch Block	19 (3.4)
Echocardiogram Features		
LVEF n=254	LVEF <20%	13 (5.1)
	LVEF 21-30%	28 (11.0)
	LVEF 31-40%	38 (14.9)
	LVEF 41-50%	39 (15.4)
	LVEF >50%	136 (53.5)
Other features n=343	Diastolic dysfunction	124 (35.1)
	Valve Dysfunction	259 (75.5)
	Aortic Dysfunction	133 (38.7)
	Mitral Dysfunction	216 (63.0)
	Pulmonary Dysfunction	18 (5.2)
	Tricuspid Dysfunction	114 (33.2)

Supplementary table S3: Electrocardiogram (ECG) and echocardiographic findings of patients with non-malignant pleural effusions related to heart failure

Chest radiograph Features, n (%)	Cardiac n=558	Renal n=64	Hepatic n=131	p value
Left	98 (17.6)	14 (21.9)	18 (13.7)	0.328
Right	192 (34.4)	23 (35.9)	91 (69.5)	<0.001
Bilateral	268 (48.2)	27 (42.2)	22 (16.8)	<0.001
Predominance (if bilateral)	n=268	n=27	n=22	
Left	76 (28.3)	8 (29.6)	8 (36.6)	0.033
Right	126 (47.0)	17 (69.3)	11 (50)	<0.001
Equal	66 (24.6)	2 (3.2)	3 (13.6)	0.007
Size Left	n=407	n=45	n=45	
Small <25%	192 (47.1)	19 (42.2)	20 (44.4)	<0.001
Moderate 25-50%	129 (31.7)	14 (31.1)	12 (26.6)	<0.001
Large 50-75%	77 (18.9)	10 (22.2)	9 (20.0)	0.061
Massive >75	9 (2.2)	2 (3.1)	4 (8.9)	0.300
Size Right	n=476	n=54	n=115	
Small <25%	188 (39.5)	17 (31.5)	30 (22.9)	0.043
Moderate 25-50%	190 (39.9)	21 (38.8)	27 (20.6)	0.010
Large 50-75%	83 (17.4)	12 (22.2)	30 (22.9)	0.074
Massive >75	15 (3.15)	4 (7.4)	28 (21.4)	<0.001

Supplementary table S4: Chest radiograph features of non-malignant pleural effusions related to heart, renal and hepatic failure.

Hepatic Effusions n=131	n (%)
Abdominal Ultrasound features	
Hepatomegaly	34 (26)
Cirrhosis	90 (68.7)
Portal Hypertension	65 (49.6)
Splenomegaly	59 (45)
Ascites	80 (61.1)
Mild	32 (24.4)
Moderate	20 (15.3)
Severe	7 (5.3)
Unclassified	21 (27.0)

Supplementary table S5: Abdominal ultrasound features of patients with non-malignant pleural effusions related to hepatic failure

Variable	HR	95% CI	p-value
Age (year)	1.013	1.002-1.024	0.020
Ischaemic Heart Disease	1.238	0.963-1.592	0.096
NT proBNP >450 pg/mL	1.508	1.191-1.911	<0.001
LVEF ≤50%	1.135	0.967-1.332	0.122
Diastolic dysfunction	0.738	0.592-0.920	0.007

Supplementary table S6: Pertinent factors associated with prognostic influence in the cardiac non-malignant pleural effusion group. LVEF – left ventricular ejection fraction

Medication	Cardiac n=560	Renal n=64	Hepatic n=131	p value
Loop Diuretic	402 (71.8)	28 (43.8)	89 (67.9)	0.060
Furosemide	385 (68.8)	28 (43.8)	84 (64.1)	0.002
Bumetanide	5 (0.9)	0	3 (2.3)	0.278
Mineralocorticoid antagonist	73 (13)	7 (10.9)	85 (64.9)	<0.001
Spironolactone	56 (10.0)	5 (7.8)	82 (62.5)	<0.001
Eplerenone	12 (2.1)	0	1 (0.8)	0.487
Beta-blocker	175 (31.8)	14 (21.9)	43 (32.8)	0.261
ACEi/ARB	161 (28.7)	10 (15.6)	9 (6.9)	<0.001

Supplementary table S7: Medications utilised in non-malignant pleural effusion management effusions (ACEi: Angiotensin Converting Enzyme Inhibitor; ARB: Angiotensin Receptor Blocker)

	Intervention	n (%)
Cardiac Effusions n=560	PPM	5 (0.9)
	ICD	6 (1.1)
	Resynchronisation device	2 (0.4)
	Single chamber	1 (0.2)
	CRTD	1 (0.2)
Renal Effusions n=64	Dialysis	7 (10.9)
	PD	1 (1.6)
	HD	6 (9.4)
Hepatic Effusions n=131	TIPSS	6 (4.6)
	Paracentesis	76 (58)

Supplementary table S8: Interventional treatments for non-malignant pleural effusions (PPM - permanent pacemaker; ICD - implantable cardiac defibrillator; CRTD - cardiac resynchronisation device and defibrillator; PD - peritoneal dialysis; HD - haemodialysis; TIPPS – transhepatic portosystemic shunt.

Hepatic NMPE	Human albumin solution given n=15	No human albumin solution given n=109
Electrolyte disturbance	7 (46.7)	19 (17.4)
AKI < 48 hrs	1 (6.7)	2 (1.8)
AKI > 48 hrs	2 (13.3)	6 (5.52)
eGFR mean (SD)	66.1 (19.7)	65.4 (22)

Supplementary table S9: Frequencies of electrolyte imbalances and acute kidney injury (AKI) in the hepatic NMPE group

Variable	Transudate (n=279)	Exudate (n=44)
Male	196 (70%)	27 (61.4%)
Median age (IQR)	79.0 (72.0-79.0)	79.0 (70.0-85.25)
Ischaemic heart disease	101 (36.2%)	14 (32.0%)
Atrial fibrillation	127 (45.5%)	18 (40.1)
Chronic kidney disease	88 (27.0%)	10 (22.7%)
Heart failure with preserved ejection fraction	99/151 (65.5%)	11/34 (32.3%)
Heart failure with reduced ejection fraction	52/151 (34.5%)	23/34 (67%)
Chest x-ray features		
Left	47 (18.8%)	10 (22.7%)
Right	115 (41.2%)	13 (29.5%)
Bilateral	163 (58.4%)	19 (43.2%)
Size Left		
Small <25%	123 (44.1%)	11 (25.0%)
Moderate 25-50%	72 (58.8%)	10 (22.7%)
Large 50-75%	39 (14.0%)	6 (13.6%)
Massive >75	3 (1.1%)	1 (2.3%)
Size Right		
Small <25%	119 (42.7%)	11 (25.0%)
Moderate 25-50%	115 (41.2%)	12 (27.3%)
Large 50-75%	43 (15.4%)	8 (18.2%)
Massive >75	7 (2.5%)	1 (2.3%)
Biochemical features		
pH median (IQR)	7.48 (7.43-7.53)	7.46 (7.41-7.51)
Glucose mmol/L median (IQR)	131.4 (115.1-162.0)	127.0 (108.0-156.6)
Pleural fluid LDH:Serum LDH ratio median (IQR)	0.356 (0.279-0.431)	0.685 (0-0.934)
Protein g/L median (IQR)	20.0 (16.0-26.0)	24.0 (16.0-32.0)
Cytological predominance		
Lymphocyte	115 (41.0%)	18 (41.0%)
Mesothelial/macrophage	140 (50.5%)	20 (45.4%
Neutrophil	28 (10.0%)	6 (13.6%)

Supplementary table S10: Comparison of features of transudative and exudative pleural effusions related to cardiac failure. IQR – interquartile range; LAD – lactate dehydrogenase
